# Association between serum resistin concentration and hypertension: A systematic review and meta-analysis

**DOI:** 10.18632/oncotarget.17561

**Published:** 2017-05-02

**Authors:** Yuxiang Zhang, Yixing Li, Lin Yu, Lei Zhou

**Affiliations:** ^1^ State Key Laboratory for Conservation and Utilization of Subtropical Agro-Bioresources, College of Animal Science and Technology, Guangxi University, Nanning, 530004, P.R. China; ^2^ The Institute for Diabetes, Obesity, and Metabolism, Perelman School of Medicine at the University of Pennsylvania, Philadelphia, PA 19104, USA; ^3^ Pharmacology Graduate Program, Perelman School of Medicine at the University of Pennsylvania, Philadelphia, PA 19104, USA; ^4^ Public Health Certificate Program, Perelman School of Medicine at the University of Pennsylvania, Philadelphia, PA 19104, USA

**Keywords:** resistin, hypertension, meta-analysis

## Abstract

**Objectives:**

Recent studies have suggested the involvement of adipokines in the pathogenesis of cardiovascular diseases, including hypertension. In this study, we evaluated the significance of serum resistin levels in hypertensive patients using a meta-analysis approach.

**Materials and Methods:**

Relevant articles were retrieved by searching the following databases: PubMed, Embase, Ovid Medline, ISI Web of Knowledge. The retrieved studies were subjected to a thorough screening procedure to identify case-control studies that contained the required data. Data were extracted from each study and analyzed by Stata software and Review Manager software. In total, 14 case-control studies, containing 718 hypertensive patients and 645 normotensive controls, were included in this study. The major result of the meta-analysis revealed a statistically significant association between serum resistin concentration and hypertension (SMD = 0.85, 95% CI: [0.15, 1.54]), and the association was more obvious in Asian and Hispanic populations, diabetic population and studies with larger size cohorts. Publication bias was a low probability event for overall comparisons.

**Conclusions:**

Based on our results, we conclude that serum resistin level in hypertensive patients is higher than normotensive controls, indicating resistin might be a risk factor for hypertension.

## INTRODUCTION

Resistin is a 12.5 kDa cysteine-rich polypeptide discovered in a screen for adipocyte gene products that are down regulated by anti-diabetic thiazolidinedione (TZD) drugs in mice [1]. In rodents, resistin is an adipokine, secreted from white adipocytes and is reported to regulate glucose metabolism and insulin sensitivity [2]. Systemic treatment or transgenic overexpression of resistin in rodents decreases the ability of insulin to suppress hepatic glucose production [3, 4]. Conversely, ablation of the retn gene or reduction in resistin protein by antisense oligonucleotide treatment improves insulin sensitivity through AMPK activation [5, 6]. Resistin is detectable in human serum, but unlike rodent resistin, human resistin is predominantly produced by macrophages [7], and as in rodents, resistin level decreases with thiazolidinedione treatment in humans [8]. Besides a role in metabolism, resistin has also been associated with cardiovascular diseases (CVD) [9–11]. High plasma resistin levels were observed in CVD patients, and its function has been related to vasodilation, and endothelial dysfunction [9, 11], indicating that resistin might be involved in the regulation of blood pressure.

Hypertension is one of the most important risk factors for coronary heart diseases and stroke, and has been associated with obesity and diabetes. Recent studies have shown that adipokines, such as adiponectin, leptin and resistin might involve in the development of hypertension [12, 13]. Many studies have investigated the correlation between circulating resistin levels and hypertension. The results of initial efforts were conflicting, with some, but not all studies identifying a significant correlation between serum resistin level and hypertension incidence. Thomopoulos and colleagues reported that increased resistin and decreased adiponectin plasma levels are asscciated with sustained and masked hypertension, and resistin is associated 2.5 times more frequently with sustained hypertension than with masked hypertension [14]. However, Olszanecka and colleagues did not find any difference in the serum resistin level between the hypertensive and normotensive pre- and post-menopausal women [15]. The discrepancy among study results could be related to differing demographics of the study groups as well as the various sample sizes. Therefore, in this study we carried out a systematic review to synthesize and analyze the published data on the association of resistin with the risk of hypertension.

## RESULTS

### Characteristics of enrolled studies

**Figure 1:**
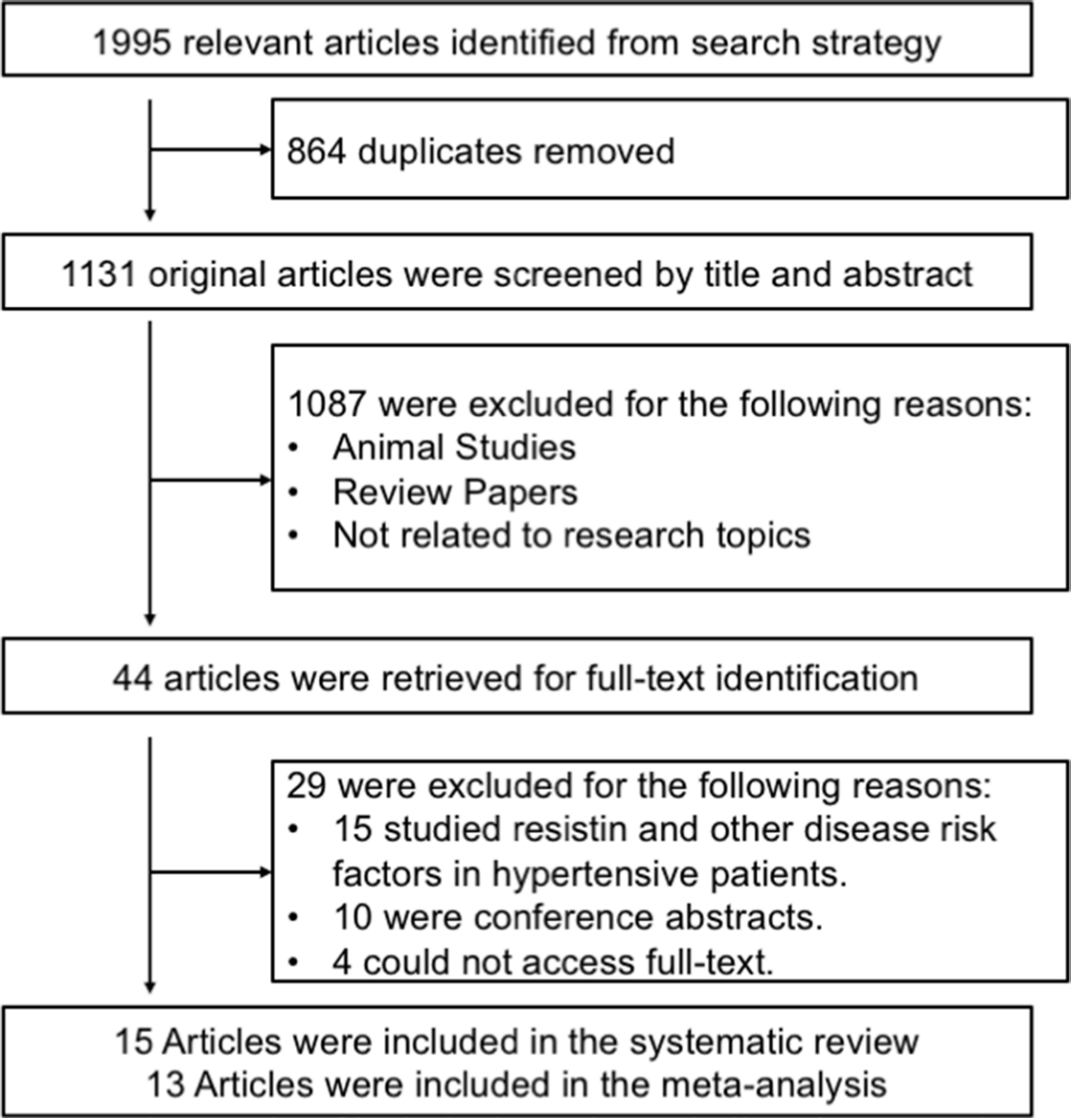
Flow chart of literature search and study selection

The database search originally resulted in retrieval of 1995 articles related the search keywords. The flow diagram of the study selection process is presented in Figure 1. Finally, 15 articles were included in the systematic review and 13 articles containing 14 case-control studies were enrolled in the meta-analysis [14–26]. The major discovery of the two studies that are excluded from the meta-analysis were summarized in Supplementary Table 2. The publication year of these studies ranged between 2003 and 2013. All articles were case-control studies reporting the relationship between serum resistin levels and hypertension in European, Asian and Hispanic populations. Table 1 displays the basic features of the studies and the detection kits used in each study. The available clinical characteristics of the enrolled studies are summarized in Table 2.

**Table 1:** Main features of included studies used in the meta-analysis

First Author and Year	Country	Study Population		ELISA Kits
		Case (*n*)	Control (*n*)	
Furuhashi M 2003	Japan	Hypertension Patients (21)	Normotensive Control (18)	Phoenix Pharmaceuticals Inc., Belmont, CA, USA
Zhang JL 2003	China	Hypertensive Type 2 Diabetic Patients (13)	Normotensive Control (12)	Phoenix Pharmaceuticals Inc., Belmont, CA, USA
Papadopoulos DP 2005	Greek	Prehypertension Patients (26)	Normotensive Control (24)	Bio Vendor Laboratory Medicine Inc., Czech Republic
Takata Y 2008	Japan	Hypertensive Type 2 Diabetic Patients (91)	Normotensive Type 2 Diabetic Patients (64)	LINCO Research Inc., St Charles, MS, USA
Papadopoulos DP 2009	Greek	Masked Hypertensive Patients (24)	Normotensive Control (106)	Bio Vendor Laboratory Medicine Inc., Czech Republic
Bo S 2009	Italy	Hypertension Patients (31)	Normotensive Control (27)	R&D Systems, Minneapolis, MN, USA
Yang J 2009	China	Hypertension Patients (137)	Normotensive Control (134)	Adlitteram Diagnostic laboratories, USA
Olszanecka A 2010_1	Poland	Hypertensive Postmenopausal Women (74)	Normotensive Postmenopausal Women (19)	Bio Vendor Laboratory Medicine Inc., Czech Republic
Olszanecka A 2010_2	Poland	Hypertensive Premenopausal Women (78)	Normotensive Premenopausal Women (21)	Bio Vendor Laboratory Medicine Inc., Czech Republic
Thomopoulos C 2011	Greek	Hypertension Patients (105)	Normotensive Control (130)	Bio Vendor Laboratory Medicine Inc., Czech Republic
Mahadik SR 2012	India	Hypertension Patients (23)	Normotensive Control (41)	LINCO Research Inc., St Charles, MS, USA
Stepien M 2012	Poland	Hypertensive Severe Obesity Patients (10)	Normotensive Simple Obesity Patients (7)	EMD Merck Millipore Corp., Germany
Rubio-Guerra AF 2013	Mexico	Hypertensive Type 2 Diabetic Patients (30)	Normotensive Non-Diabetic Subjects (30)	R&D Systems, Minneapolis, MN, USA
Stepien M 2014	Poland	Hypertension Patients (55)	Normotensive Patients (12)	DRG Instruments GmbH, Germany

**Table 2:** Clinical measurements available in all the studies included in the meta-analysis

First Author and Year	Age (years)		Gender (M|F)		BMI (Kg/m^2^)		Systolic/Diastolic Blood Pressure (mmHg)	
	Case	Control	Case	Control	Case	Control	Case	Control
Furuhashi M 2003	47.2 ± 11.7	45.9 ± 13.7	8|13	10|8	24.1 ± 2.8	24.2 ± 3.1	NA/ NA	NA/NA
Zhang JL 2003	60.6 ± 7.2	49.1 ± 7.5	6|7	5|7	29.1 ± 4.3	22.7 ± 1.9	159.1 ± 10.3/ 95.7 ± 6.2	108.7 ± 9.2/70.6 ± 8.9
Papadopoulos DP 2005	52 ± 5	53 ± 6	14|12	13|11	23 ± 1.5	23.2 ± 1.4	133 ± 2/ 87 ± 2	116 ± 3/76 ± 2
Takata Y 2008	63.8 ± 10.4	57.3 ± 13.9	51|40	37|27	25.4 ± 4.0	25.4 ± 5.8	140.9 ± 18.9/ 79 ± 9.5	120.5 ± 12.5/73.3 ± 7.0
Papadopoulos DP 2009	46 ± 7	44 ± 6	11|13	52|54	25.9 ± 2.1	25.5 ± 2.4	138 ± 6/ 90 ± 4	122 ± 7/79 ± 4
Bo S 2009	51.1 ± 5.4	50.8 ± 4.6	NA	NA	27.9	23.9 ± 3.5	NA/ NA	NA/NA
Yang J 2009	54.1 ± 8.1	53.0 ± 7.7	95|42	68|66	25.60 ± 3.19	24.00 ± 2.73	140.34 ± 10.47/ 92.69 ± 7.19	122.37 ± 11.35/79.16 ± 7.43
Olszanecka A 2010_1	51.9 ± 2.5	55.6 ± 3.2	0|74	0|19	27.1 ± 3.3	25.7 ± 3.5	132.8 ± 6.3/ 81.8 ± 8.7	111.9 ± 7.3/70.7 ± 5.2
Olszanecka A 2010_2	50.1 ± 2.8	46.3 ± 2.7	0|78	0|21	27.0 ± 3.1	24.6 ± 4.7	133.4 ± 8.1/ 82.5 ± 6.7	113.8 ± 5.0/71.0 ± 4.3
Thomopoulos C 2011	47 ± 6	48 ± 7	51|54	74|56	26.8 ± 4	26.4 ± 3.9	146 ± 4/ 91 ± 4	126 ± 5/80 ± 4
Mahadik SR 2012	47.96 ± 1.45	47.2 ± 1.31	13|10	21|20	22.5 ± 0.33	22.0 ± 0.30	136.7 ± 4.19/ 86.7 ± 2.91	115.6 ± 2.45/75.8 ± 1.21
Stepien M 2012	54.30 ± 12.09	46.57 ± 13.58	2|8	3|4	38.51 ± 2.96	32.49 ± 2.18	NA/ NA	NA/NA
Rubio-Guerra AF 2013	60 ± 9	58 ± 11	16|14	19|11	30.4 ± 5	29.8 ± 6	175/ 93	122/76
Stepien M 2014	59.3 ± 7.4	46.5 ± 12.2	20|35	5|7	36.1 ± 5.5	34.1 ± 2.60	139.8 ± 21.2	121.9 ± 13.9

### Association between the resistin serum levels and hypertension

**Figure 2:**
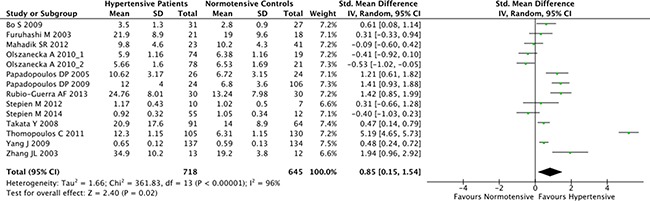
Forest plot of the differences in serum resistin levels between hypertensive patients and healthy controls Abbreviations: 95% CI, 95% confidence interval.

**Figure 3:**
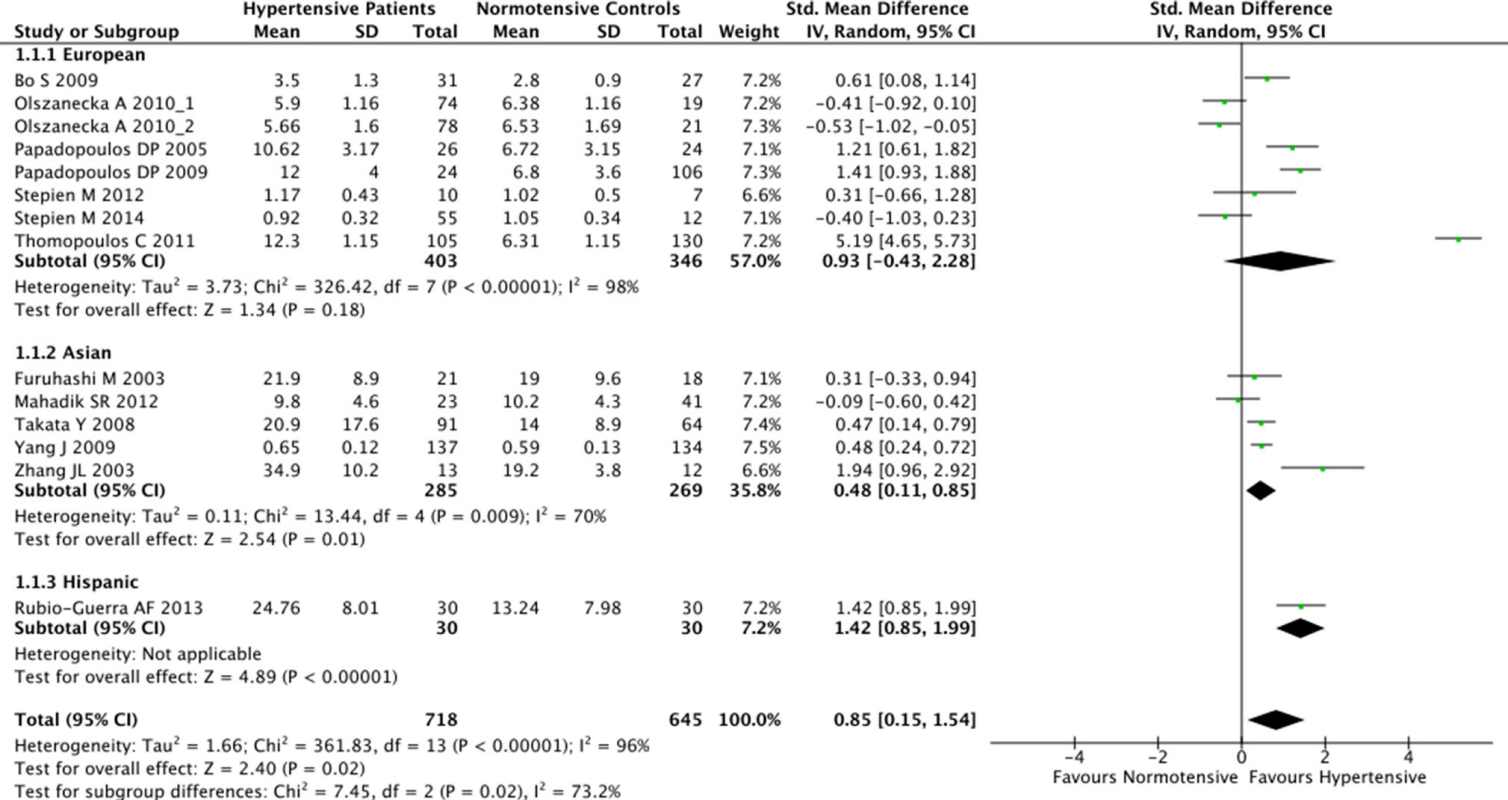
Subgroup analyses for the differences of serum resistin levels between hypertensive patients and healthy controls in different ethnicities Abbreviations: 95% CI, 95% confidence interval.

**Figure 4:**
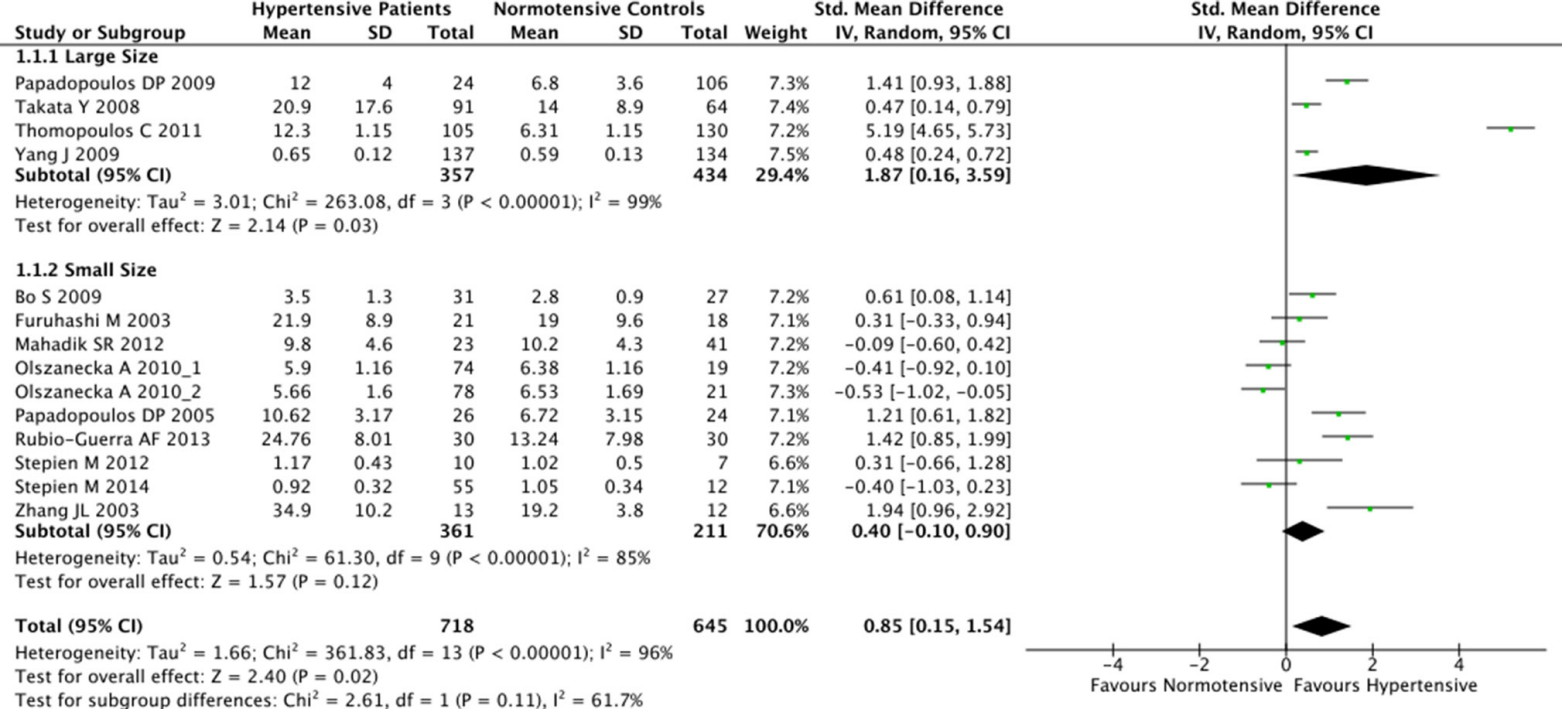
Subgroup analyses for the differences of serum resistin levels between hypertensive patients and healthy controls in studies of different sizes Studies with total sample size over 100 were considered as large size studies, and studies with a sample size less than 100 were considered as small size studies. Abbreviations: 95% CI, 95% confidence interval.

The overall result of the correlation between resistin levels and hypertension is shown in Figure 2. The random-effect model was applied due to existence of heterogeneity among the studies (*P* < 0.001). A positive association between resistin serum levels and hypertension was identified in this meta-analysis (SMD = 0.85, 95% CI: 0.15–1.54, *P* = 0.02). Subgroup analysis based on the ethnicity revealed that serum resistin levels were significantly higher in hypertensive patients, compared to healthy controls, in both Asian (SMD = 0.48, 95% CI: 0.11–0.85, *P* = 0.01) and Hispanic populations (SMD = 1.42, 95% CI: 0.85–1.99) (Figure 3). The serum resistin trended towards a higher level in European hypertensive patients than the normotensive controls (SMD = 0.93, 95% CI: −0.43–2.28, *P* = 0.18) (Figure 3). In addition, resistin level seems to have a stronger correlation with hypertension in diabetic patients, since the subgroup analysis of the diabetic population showed higher standard mean differences in the hypertensive and normotensive population in the diabetic population (SMD = 1.20, 95% CI: 0.32–2.07, *P* = 0.008) than the non-diabetic population (SMD = 0.74, 95% CI: −0.14–1.62, *P* = 0.10) (Supplementary Figure 1). However, the number of the studies on diabetic population was limited, and thus future studies are needed to confirm this finding. Further, subgroup analysis by sample size indicated that the association between serum resistin level and hypertension were more prominent in the large sample size studies (total study size over 100, SMD = 1.87, 95% CI: 0.16–3.59, *P* = 0.03) than in the small size studies (total study size less than 100, SMD = 0.40, 95% CI: −0.10–0.90, *P* = 0.12) (Figure 4).

### Sensitivity analysis and publication bias

**Figure 5:**
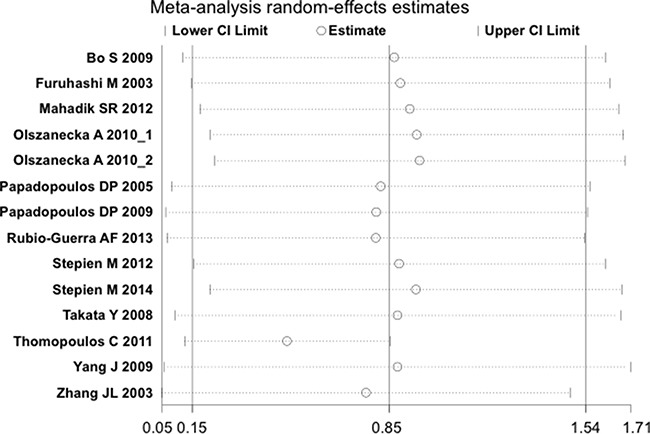
Sensitivity analysis plot of the differences in serum resistin levels between hypertensive patients and healthy controls Meta-analysis random-effects estimates were used. The two ends of he dotted lines represented the 95% CI.

**Figure 6:**
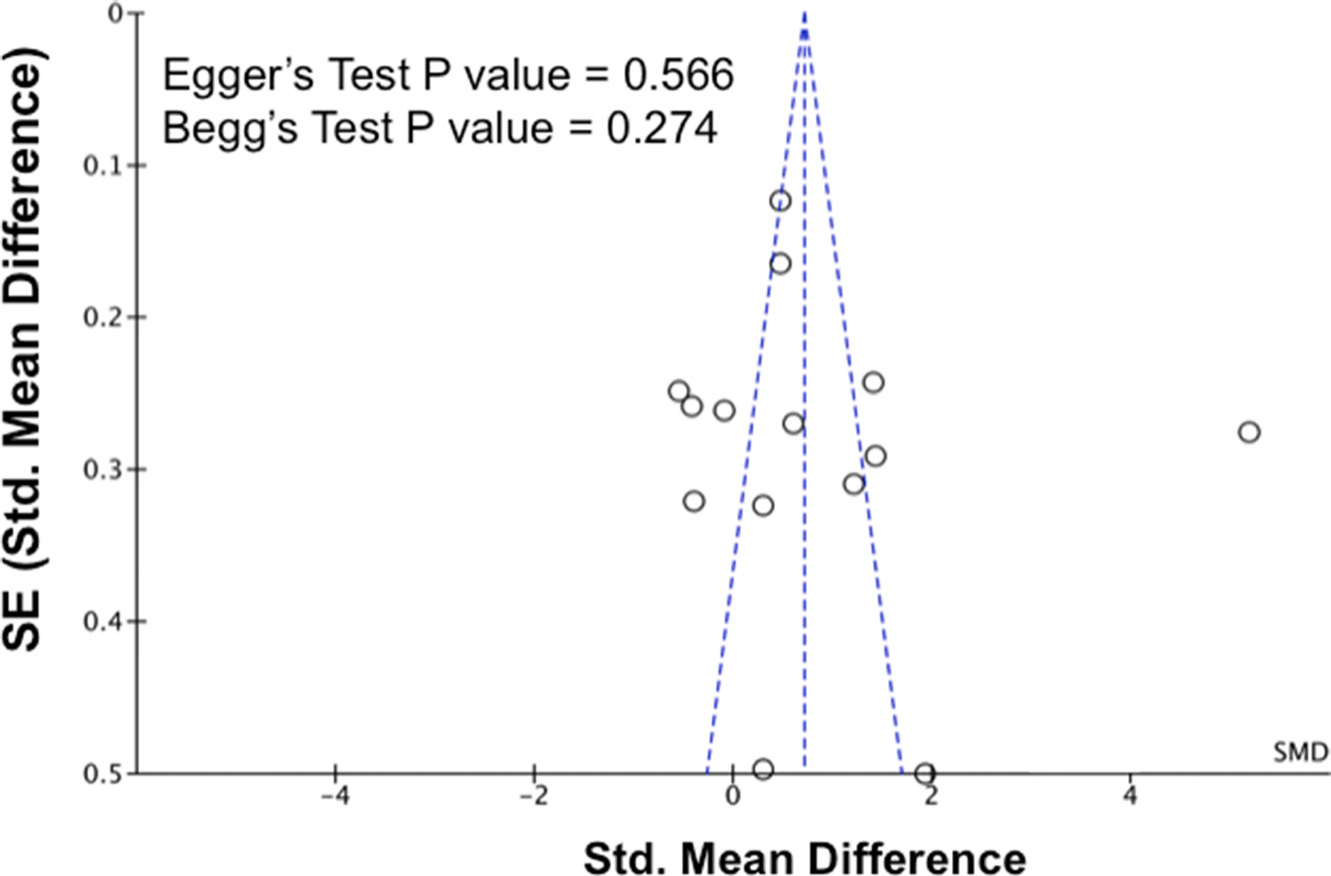
Publication biases on the differences of serum resistin levels between hypertensive and normotensive individuals The *P* values for Egger's test and Begg's test are shown in the figure. Abbreviations: 95% CI, 95% confidence interval; SE, standard error.

The removal of any single study in sensitivity analysis did not change the overall statistical significance, suggesting that this meta-analysis is relatively stable and reliable (Figure 5). Additionally, Egger's test and Begg's test for funnel plot asymmetry showed no significant publication bias among the studies (Figure 6).

### Random-effects meta-regression analysis

In order to look for additional sources of heterogeneity, meta-regression analyses were conducted by incorporating sample size, mean difference of age, difference of gender ratio, and mean difference of body mass index as covariates, while none of them exhibited an obvious confounding influence on the association between the serum resistin levels and hypertension risk (Table 3).

**Table 3:** Meta-regression analysis coefficients for serum resistin level in the examined group of studies

Variables	Coefficient (SE)	95% Confidence Interval	*P*
Sample Size	0.01 (0.01)	[−0.01, 0.02]	0.18
Mean Difference of Age	−0.05 (0.10)	[−0.27, 0.16]	0.59
Difference of Male %	−2.13 (4.49)	[−12.01, 7.76]	0.65
Mean Difference of BMI	−0.07 (0.21)	[−0.53, 0.38]	0.73

## DISCUSSION

In our analysis, fourteen case-control studies investigating the correlation between serum resistin concentration and hypertension were included. Ten of those studies reported higher resistin level in the hypertension patients, and seven of them were statistically significant. Collectively, our meta-analysis showed that resistin concentration of hypertensive patients was significantly higher than that of normal controls (SMD [95% CI], 0.85 [0.15, 1.54]), indicating that resistin might be a potential risk factor and biomarker for hypertensive patients.

Resistin in human is mainly produced by the macrophages, and could exert effects on several tissues. To elucidate the mechanisms of resistin regulation in each tissue, several potential resistin receptors have been suggested, including Toll-like Receptor 4 (TLR4) [27, 28], an isoform of decorin (DCN), mouse receptor tyrosine kinase-like orphan receptor 1 (ROR1), and adenylyl cyclase-associated protein 1 (CAP1). Since resistin is involved in the regulation of different processes in different cell types and those receptors differ in their tissue distribution, it is possible different receptors could be involved in mediating its numerous effects in various tissues. It has been reported that resistin could regulate metabolic process and adipogenesis in 3T3-L1 cells through its binding to ROR1 and DCN [29, 30]. On the other hand, resistin could bind to human leucocytes through its interaction with TLR4 and activate pro-inflammatory pathways and cytokine expression [28]. In addition, resistin could also bind to CAP1 in monocytes, whose intracellular signaling pathway could modulate inflammatory action of monocytes [31]. These two receptors might involve in resistin regulation of pro-inflammatory process and its association with cardiovascular diseases [32].

The mechanism underlying the association between the resistin and hypertension still remains to be elucidated. One possible mechanism might be mediated via the TLR4. It has been reported that resistin could induces hypertension in wild type (WT) mice by activating the renin-angiotensin system through up-regulation of *Agt* expression in the liver [13]. These phenotypes induced by resistin administration were not observed in the *Tlr4*^−/−^ mice, indicating the regulation of blood pressure by resistin is TLR4-dependent. Another potential mechanism is that resistin could reduce endothelial nitric oxide synthase expression, and elevate ET-1 expression as well as its release in human endothelial cells [33, 34]. This could also explain the association between the resistin and hypertension.

In order to consider other factors that may affect the link between serum resistin level and hypertension, we performed a stratified analysis based on ethnicity and sample size. Subgroup analysis showed that the correlation between the resistin and hypertension is more consistently reported in Asian and Hispanic populations. In European hypertensive patients, resistin still trended toward a higher level than the normotensive controls, although the results are more heterogeneous. In addition, the studies with larger cohort size showed more significant correlation between resistin and hypertension patients than the small sized studies. Furthermore, several other potential confounding factors have also been analyzed in this study by the meta-regression methods. Still, none of them were correlated with the level of serum resistin, indicating those factors are not likely to explain the differences in the resistin levels between hypertensive patients and normotensive controls.

Several limitations should be acknowledged for the current meta-analysis. First, heterogeneity is a chief issue when analyze data from different studies, especially with limited number of studies. Although we have used subgroup and meta-regression analyses to evaluate potential sources of heterogeneity, the heterogeneity from other sources might still interfere with our analyses and conclusions. In addition, in our subgroup analysis of the diabetic and non-diabetic populations, some studies in non-diabetic group did not report clearly whether the patients have defects in glucose metabolism, which could interfere with our interpretation of the result. Second, due to unavailability of the data, only four potential confounding factors were analyzed by meta-regression analyses. Other factors, such as insulin resistance, glucose tolerance, blood glucose level, body fat composition, and others, were not analyzed since few studies reported those clinical measurements. Future studies with appropriate controls and more detailed measurement for those factors might be needed to address those issues. Third, our search strategy was limited to the paper published in English and Chinese from the four major publication databases. Articles in other languages and deposited in other databases were not considered, which is susceptible to a literature bias. Last but not least, the studies used in this analysis were all case-control studies, which may prove an association but do not demonstrate causation between the resistin and high blood pressure. It still remains to be shown whether high resistin level contributes to hypertension development, or high blood pressure induces resistin expression in human population by other types of study design.

Taken together, the present study revealed higher serum resistin levels in hypertensive patients compared to healthy controls, indicating that elevated serum resistin levels correlated with hypertension development. Future studies with a larger population and better study design are much needed to confirm this finding, and decide whether resistin may be of clinical value in the treatment of hypertensive patients.

## MATERIALS AND METHODS

### PRISMA guideline

This systematic review and meta-analysis was performed according to the checklist and guidelines from the PRISMA (the preferred reporting items for systematic reviews and meta-analyses) statement [35], as presented in the Supplementary Table 1.

### Search strategy

Published case-control studies were identified via comprehensive search (last search Feb 7th, 2017) of PubMed, Embase, Ovid Medline, ISI Web of Knowledge. The studies reporting the association between serum Resistin levels and hypertension were identified from the databases by utilizing the search terms (“Resistin” or “RETN” or “RELM” or “FIZZ” or “Adipose tissue-specific secretory factor” or “XCP1”) and (“hypertension” or “hypertensive” or “blood pressure”). Additionally, manual searches were employed to identify potentially relevant articles from cross-references of important studies.

### Study selection

Randomized case-control studies investigating the association between serum resistin levels and hypertension were considered for this meta-analysis. Duplicate studies or studies lacking complete data were excluded. Only the most complete or the most recent study was enrolled for the duplicate studies.

### Data extraction

To minimize bias and improve the reliability, two investigators independently collected information based on the selection criteria and reached a consensus on all items after discussion and re-examination. The following relevant data were extracted from the eligible studies: surname of first author, year of publication, source of publication, study type, study design, source of publication, sample size, age, gender, ethnicity and country of origin, detection method of serum resistin serum levels, and resistin expression levels. For the studies that only reported median and interquartile range, medians and interquartile ranges were converted to mean and standard deviation according to the method published previously [36]. All authors agreed with the final enrolled studies.

### Statistical analysis

To provide quantitative evidence and minimize variance of the summary, this meta-analysis was performed by applying random-effect model or fixed-effect mode. When heterogeneity existed among studies, a random-effect model was used; otherwise a fixed-effect model was utilized. We used standard mean difference method to compare the case and control group difference in serum resistin level. Random-effect meta-regression analysis was used to analyze correlation between resistin level and potential confounding factors, including sample size, differences in male percentages, mean difference of age and BMI. Egger's test and Begg's test was used to examine the publication bias in the funnel plots. All statistical analyses were conducted with the usage of STATA software version 14.2 (Stata Corp, College Station, TX, USA) and Review Manager 5.3 (The Cochrane Collaboration).

## SUPPLEMENTARY MATERIALS FIGURES AND TABLES





## Abbreviations

95% CI: 95% Confidence Interval
CAP1: Cyclase-Associated Protein 1
CVD: Cardiovascular Diseases
PRISMA: Preferred Reporting Items for Systematic Reviews and Meta-Analyses
ROR1: Receptor Tyrosine Kinase-Like Orphan Receptor 1
SMD: Standard Mean Difference
TLR4: Toll-Like Receptor 4
