# Supplementary material for: Association between serum resistin concentration and hypertension: A systematic review and meta-analysis
Source: Oncotarget. 2017 May 2;8(25):41529–37. doi: 10.18632/oncotarget.17561 (PMC5522312; doi:10.18632/oncotarget.17561)
Supplement: Supplementary file 1 [file oncotarget-08-41529-s001.pdf]

Association between serum resistin concentration and hypertension: A systematic review and meta-analysis

Supplementary Materials

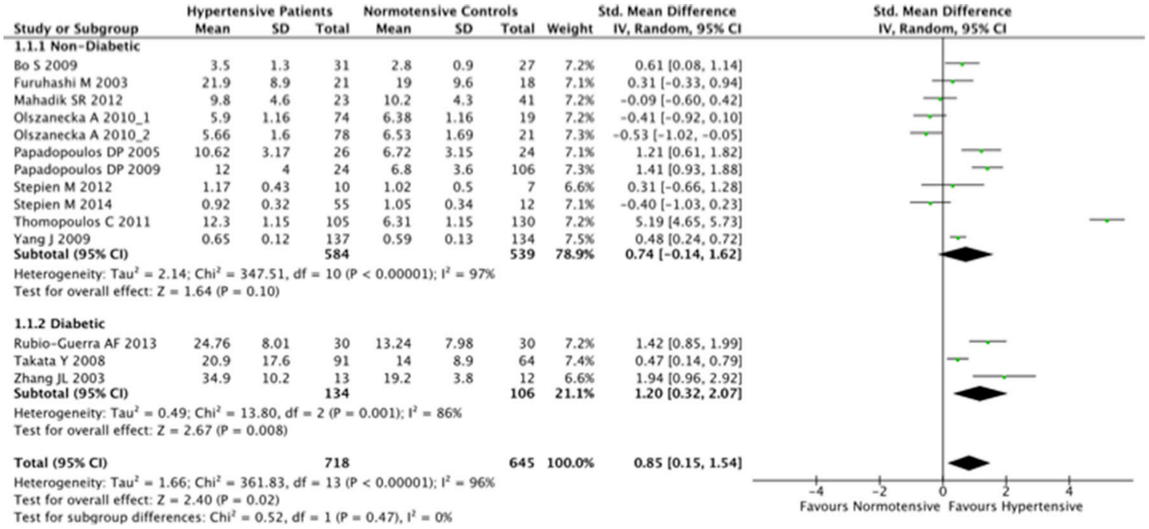

Supplementary Figure 1: Subgroup analyses for the differences of serum resistin levels between hypertensive patients and healthy controls in diabetic population and non-diabetic population. Abbreviations: 95% CI, 95% confidence interval.

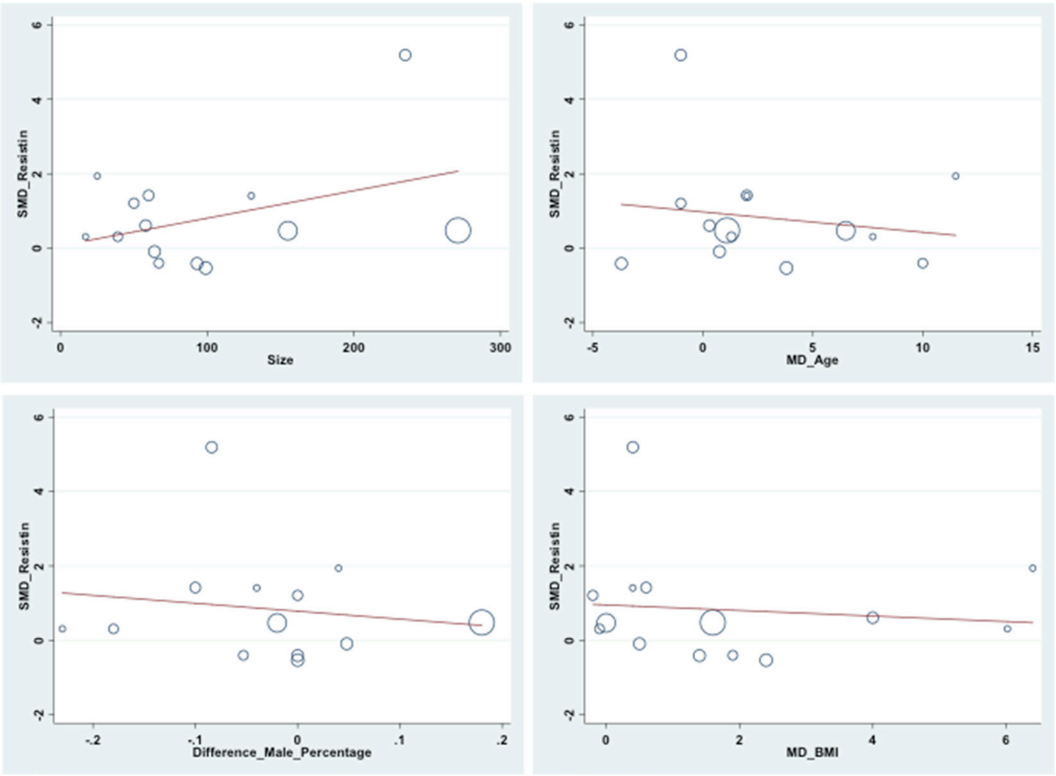

Supplementary Figure 2: Regression plot for the meta-regression analysis shown in Table 3. Abbreviations: SMD, standard mean difference; MD, mean difference.

Supplementary Table 1: PRISMA 2009 Checklist. See_Supplementary_Table_1

Supplementary Table 2: Summary of studies addressing the association between resistin and hypertension that were excluded from our meta-analysis

First Author	Title	Journal	Year	Study Design	Major Discovery	Reasons of not included in the meta- analysis
Papadopoulos DP	Adiponectin--insulin and resistin plasma levels in young healthy offspring of patients with essential hypertension	Blood Press	2008	Offsprings of Case Control Study	Increased blood pressure and serum resistin levels pre-exist in young healthy offspring with positive family history for essential hypertension.	This is a study of the offspring of the hypertensive patients
Niaz S	Serum Resistin Levels in Patients of Hypertension and Coronary Artery Disease	Pakistan Journal of Medical and Health Sciences	2016	Comparative Study	Serum resistin levels were significantly raised in patients of hypertension and coronary artery increasing severity of cardiac disease.	Values of data were not complete for data extraction. Results were presented in bar graph.
